# Automated Identification of Skull Fractures With Deep Learning: A Comparison Between Object Detection and Segmentation Approach

**DOI:** 10.3389/fneur.2021.687931

**Published:** 2021-10-29

**Authors:** Wei Shan, Jianwei Guo, Xuewei Mao, Yulei Zhang, Yikun Huang, Shuai Wang, Zixiao Li, Xia Meng, Pingye Zhang, Zhenzhou Wu, Qun Wang, Yaou Liu, Kunlun He, Yongjun Wang

**Affiliations:** ^1^Department of Neurology, Beijing Tiantan Hospital, Capital Medical University, Beijing, China; ^2^National Center for Clinical Medicine of Neurological Diseases, Beijing, China; ^3^Beijing Institute for Brain Disorders, Beijing, China; ^4^Department of Orthopedics, The Affiliated Hospital of Qingdao University, Qingdao, China; ^5^Shandong Key Laboratory of Industrial Control Technology, School of Automation, Qingdao University, Qingdao, China; ^6^Laboratory of Translational Medicine, Chinese PLA General Hospital, Beijing, China; ^7^Key Laboratory of Ministry of Industry and Information Technology of Biomedical Engineering and Translational Medicine, Chinese PLA General Hospital, Beijing, China

**Keywords:** skull fractures, deep learning algorithms, automated detection, CT bone algorithm sequences, retrospective study

## Abstract

**Objective:** Skull fractures caused by head trauma can lead to life-threatening complications. Hence, timely and accurate identification of fractures is of great importance. Therefore, this study aims to develop a deep learning system for automated identification of skull fractures from cranial computed tomography (CT) scans.

**Method:** This study retrospectively analyzed CT scans of 4,782 patients (median age, 54 years; 2,583 males, 2,199 females; development set: *n* = 4,168, test set: *n* = 614) diagnosed with skull fractures between September 2016 and September 2020. Additional data of 7,856 healthy people were included in the analysis to reduce the probability of false detection. Skull fractures in all the scans were manually labeled by seven experienced neurologists. Two deep learning approaches were developed and tested for the identification of skull fractures. In the first approach, the fracture identification task was treated as an object detected problem, and a YOLOv3 network was trained to identify all the instances of skull fracture. In the second approach, the task was treated as a segmentation problem and a modified attention U-net was trained to segment all the voxels representing skull fracture. The developed models were tested using an external test set of 235 patients (93 with, and 142 without skull fracture).

**Results:** On the test set, the YOLOv3 achieved average fracture detection sensitivity and specificity of 80.64, and 85.92%, respectively. On the same dataset, the modified attention U-Net achieved a fracture detection sensitivity and specificity of 82.80, and 88.73%, respectively.

**Conclusion:** Deep learning methods can identify skull fractures with good sensitivity. The segmentation approach to fracture identification may achieve better results.

## Introduction

Head trauma is one of the most common diseases observed in emergency departments. Cases of head trauma caused by instances of relatively high force, such as motor vehicle accidents, pedestrian injuries, falls, and assault commonly present with skull fractures (1). Skull fractures can result in numerous critical and life-threatening complications, including intracranial and orbital injuries, cerebrospinal fluid (CSF) leakage, cranial nerve palsies, and vascular injuries (2). Therefore, timely and accurate diagnosis of skull fracture is very important for the management of traumatic head injury.

Cranial computed tomography (CT) is the commonly used diagnostic tool in the care of suspected skull injuries. In the present clinical practice, radiologists assess the CT scans for the presence of skull fractures. However, on the CT images, skull fractures are generally observed as very small sized narrow slits in the cranium and are present at diverse locations in diverse forms (linear, depressed, diastatic, or basilar) (3–5). Also, skull fractures, especially linear fractures, may be missed when they are within the plane of the image reconstruction (6). Furthermore, other skull features like artery entrapment gap, emissary veins, and cranial sutures share a similar appearance as that of skull fractures. These characteristics make the manual identification of skull fractures a time-consuming, laborious, and error-prone process. Therefore, an automated system for the identification of skull fractures can significantly reduce the diagnostic time and help in better management of traumatic head injury. Furthermore, such a system can aid in prioritizing the skull fracture patients for radiological assessments and further treatments.

A few studies have attempted the automatic detection of skull fractures from the CT scans using the classical methods of image manipulations like entropy function, Sobel edge detection, and selective black hat transform (7–9). However, these methods only considered local features for the prediction of skull fracture, were tested with significantly smaller datasets, and experienced a very high number of false detections. Different from the classical approach, a data-driven approach of deep learning, which is a branch of artificial intelligence (AI), has achieved remarkable progress in image interpretation tasks (10, 11). Deep learning extracts features of images through a cascade of many layers of non-linear processing units and tries to explain the representations of the image data based on the learning of multiple levels of features. Since 2012, deep learning has rapidly become the cutting-edge method in image analysis with the use of convolutional neural networks (CNNs). There has been increasing interest in the application of deep learning in medical image analysis in certain fields, including the automated analysis of diabetic retinopathy (12), mammographic lesions (13), lung nodules (14, 15), pulmonary tuberculosis (16), gastric cancer (17), and dermatological diseases (18–22). Considering its success in the medical domain (12–25), one study has also applied deep learning for the task of skull fracture identification (26). However, the achieved detection accuracy was limited, possibly due to the lack of a large amount of training data (26).

In this study, we aim to develop two different approaches of deep learning for the identification of skull fracture from the CT scans using a significantly large dataset. In the task of fracture detection, identification of the presence of the fracture and its approximate location are sufficient in clinical use. Therefore, in the first approach, we treat the task of fracture detection as an object detection problem and apply the YOLOv3 object detection algorithm for the detection of fractures. In the second approach, we employ more stringent criteria and aim to precisely segment the fractured pixels using a modified attention U-net architecture. We compare the results of these two approaches.

## Materials and Methods

### Standard Protocol Approvals, and Patient Consents

This study was approved by the Ethics Committee of the Beijing Tiantan Hospital and was in accordance with the Helsinki Declaration.

### Study Design and Participants

This study retrospectively analyzed the data from 4,782 patients admitted to the Tiantan hospital, Bejing, China from September 2016 to September 2020 with a diagnosis of skull fractures. The patients with a mention of a skull fracture in the electronic health records were reviewed by experienced clinicians and the patients with a confirmed diagnosis of skull fracture were included in this study. The patients were reviewed for the availability of good-quality cranial CT images. The data was randomly divided into a training dataset (*n* = 4,168, ~85%) and an internal test dataset (*n* = 614, ~15%) for the development and testing of the developed models. In addition to the data from patients with a skull fracture, to control for cases of false detection, we also included CT imaging data from 7,856 healthy patients in the training dataset. Lastly, for independent assessment of the model performance, an independent test dataset of 235 patients was prospectively collected and it contained CT images of 93 people with skull fractures and 142 healthy people.

### CT Acquisition and Manual Annotation of Skull Fractures

The cranial CT images were acquired according to the standard clinical CT acquisition protocol for each patient. Each CT scan contained 32–40 number of axial slices and the sagittal and coronal view spacing were between 0.43 and 0.9 (equal along both the planes). All the collected CT scans were manually labeled by a team of seven radiologists. For each CT scan, the skull fractures were labeled in the 2D axial slices in two different ways. In the first method, all the pixels representing the skull fracture were labeled as lesions (segmentation mask). In the second method, all the isolated instances of skull fracture were annotated with rectangular bounding boxes that enclose each skull fracture. In this method, each isolated fracture was identified using the coordinates (x, y), and height and width (h, w) of its bounding box within the 2D axial slices. A few examples of these manual annotation methods are presented in study. All the annotations were reviewed by two neurologists with more than 15 years of experience. Final adjudications were defined as the ground truth.

### CT Preprocessing

Prior to the analysis with the deep learning methods, all the axial 2D slices were resampled to uniform axial dimensions of 512 ×512 pixels using bilinear interpolation.

### Fracture Identification With Object Detection Approach

In the first approach, the problem of automatic identification of skull fractures was formulated as an object detection problem with isolated instances of skull fracture being the object of interest. In this approach, the aim was to correctly identify the presence of skull fracture and find its approximately accurate location in 2D CT slices. Here, each fracture was represented by a rectangular bounding box at coordinate locations x, y, and of size h, w, which tightly encloses the fracture. For each fracture, the objective of the deep learning system was to correctly predict the coordinate locations and size of the fracture bounding box (x,y,h,w) and identify the content of the box to be a fracture. The bounding boxes predicted by the deep learning system were considered to be accurate if the mean intersection over union (mIOU) between the predicted box and ground truth box was higher than 0.4.

For the identification of skull fractures with the object detection approach, we employed a YOLOv3 architecture, which is one of the most successful object detection frameworks designed for the analysis of natural images. As presented in the manuscript, the YOLOv3 architecture primarily consists of two parts; a Darknet53 backbone network and a multiscale prediction head module. The Darknet53 backbone is a stack of 53 convolution layers that acts as a feature extractor and is designed to learn abstract higher dimensional features from the input images. The multiscale prediction head module is a set of three parallel convolution blocks that process the features encoded by the Darknet53 at 3 different spatial resolutions and predict the coordinates of multiple bounding boxes (x,y,w,h), probabilities of each bounding box to contain any object (pc, pc is the confidence of bounding boxes predicted by YOLOv3.), and the probabilities of the object in each box belonging to a particular class of interest (pci, pci is the probability of the bounding boxes belongs to the i-th category.). In this study, we repurposed the YOLOv3 architecture to accept the preprocessed 2D axial CT slices as an input [input shape: (512, 512, 1)] and to predict the bounding box locations and the probability of the presence of the fracture in the bounding box as an output.

The YOLOv3 architecture was trained with the composite YOLOv3 loss function with a batch size of 8 using the Adam optimizer. Image augmentation strategies including resize, crop, zoom, horizontal flip, and rotation were randomly applied during the training process to improve the model generalization performance. The initial learning rate was set to 10^∧^{-3} and the learning rate was reduced by a factor of 2 if the loss on the validation set (20% of the training data) did not decrease for 10 consecutive epochs. The training was stopped when the validation set loss did not decrease for 50 consecutive epochs and the model with the lowest validation set loss was selected as a final skull fracture object detection model. This model was used to detect the skull fractures on the internal and external test datasets.

The performance of the YOLOv3 architecture was evaluated using precision and recall in correctly identifying the fracture bounding boxes. The box-wise precision was defined as the number of correctly detected fracture bounding boxes divided by the total number of predicted bounding boxes. Similarly, the box-wise recall was defined as the number of correctly detected fracture bounding boxes divided by the total number of ground truth fracture bounding boxes. The precision and recall were separately calculated for every patient and were averaged across all the patients to compute the dataset level performance.

### Fracture Identification With a Segmentation Approach

In the second method, we approach the problem of skull fraction identification as a lesion segmentation problem wherein the objective is to identify all the pixels containing skull fracture in the 2D CT axial slices. This is a more stringent criterion than the object detection approach and it seeks the exact location of the skull fracture. Moreover, the small size and highly heterogeneous location of skull fractures make the segmentation task more difficult.

To segment skull fractures, a modified attention U-Net architecture was used and it is presented in the manuscript. This architecture primarily consists of the encoder and decoder path. The encoder was composed of 3 residual convolution blocks which were combined with a max-pooling operation. Similarly, the decoder consisted of 3 corresponding residual attention blocks with up-sampling layers and skip connections. The architecture was designed to accept preprocessed 2D axial CT slices as an input [input shape: (512, 512, 1)] and predicted a lesion mask of size (512, 512, 1), classifying each pixel to either skull fracture or background class.

The modified attention U-Net architecture was trained in a similar manner as that of the YOLOv3. Image augmentation strategies including resize, crop, zoom, horizontal flip, and rotation were randomly applied during the training process to improve the model generalization performance. The initial learning rate was set to 10^∧^{-3} and was reduced by a factor of 2 if the dice coefficient on the validation set (20% of the training data) did not increase for 3 consecutive epochs.

For the training of the modified attention U-Net we used a composite loss function which was weighted sum of dice loss ($\sqrt{l_{dice}}$) and weighted binary cross-entropy loss ($\sqrt{l_{wBCE}}$). The total loss was defined as:


$$\begin{array}{l} \sqrt{loss=l=w_{dice}l_{dice}+w_{wBCE}l_{wBCE}} \end{array}$$

The $\sqrt{w_{dice}}$, and $\sqrt{w_{wBCE}}$ determined the relative weight of the dice and the BCE loss, and they were set to 2.0, and 1.0, respectively, in this work. Furthermore, owing to the very small size of the skull fractures relative to the background pixels, we differently defined the weight pattern of the BCE loss. The $\sqrt{l_{wBCE}}$ was defined as:


$$\begin{array}{l} \sqrt{l_{wBCE}=\overset{P}{\underset{p=0}{\sum }}W_{p}C\left(I_{p},{I^{\prime }}_{p}\right)} \end{array}$$

Where, $\sqrt{C\left(I_{p},{I^{\prime }}_{p}\right)}$ is a cross-entropy loss at pixel *p* (Ip and Ip' stand for corresponding value of pixels in groundtruth and prediction, respectively), and $\sqrt{W_{p}}$ is the weight assigned to that pixel. Here, we define the $\sqrt{W_{p}}$ to give higher weights to the pixels with a skull fracture and which are at the boundary of the skull fracture. It is calculated as:


$$\begin{array}{l} \sqrt{W_{p}=\frac{\sum Pool\left(\overset{\prime }{\underset{i}{I}}\right)}{\sum 5\times e^{-6\times \left[Pool\left(I_{i}^{\prime }\right)-1\right]}}} \end{array}$$

where $\sqrt{Pool}$ is the average pooling function, the pooling size of which is 5.

In this manner, the model was trained, and the training was stopped when the validation set dice coefficient did not increase for 50 consecutive epochs. The model with the highest validation set dice coefficient was selected as a final skull fracture segmentation model. This model was used to detect the skull fractures on the internal and external test datasets.

The performance of the segmentation model was evaluated using lesion-wise precision and recall. A continuous skull fracture lesion was considered to be correctly predicted if more than 50% of pixels in the lesion were identified as lesioned by the segmentation model. Using this criterion, lesion-wise precision was defined as the number of correctly detected skull fracture lesions divided by the total number of predicted lesions. Similarly, the lesion-wise recall was defined as the number of correctly detected lesions divided by the total number of ground truth lesions. The precision and recall were separately calculated for every patient and were averaged across all the patients to compute the dataset level performance. We selected the lesion-wise statistics rather than commonly used pixel-wise statistics because these metrics were more closely comparable to the box-wise performance assessment of the first approach. The pixel-wise precision and recall were also computed in a similar manner.

### Statistical Analysis

Model performance was compared using a Fisher exact analysis. *P* <0.05 was considered a significant difference. The statistical analysis was performed using SPSS software (version 20.0).

We assume that the detection (segmentation) results can be expressed as a confusion matrix:


$$\begin{array}{l} \left(\frac{TP}{FN}\frac{FP}{TN}\right) \end{array}$$

The boxes level metrics is defined as:


$$\begin{array}{l} precision=\frac{TP}{TP+FP}\\ recall=\frac{TP}{TP+FN} \end{array}$$

The slices level metrics is defined as:


$$\begin{array}{l} precision=\frac{1}{\left|s\right|}\underset{s\in S}{\sum }\frac{TP_{s}}{TP_{s}+FP_{s}}\\ recall=\frac{1}{\left|s\right|}\underset{s\in S}{\sum }\frac{TP_{s}}{TP_{s}+FN_{s}} \end{array}$$

The patient level metrics is defined as:


$$\begin{array}{l} precision=\frac{1}{\left|P\right|}\underset{p\in P}{\sum }\frac{TP_{p}}{TP_{p}+FP_{p}}\\ recall=\frac{1}{\left|P\right|}\underset{p\in P}{\sum }\frac{TP_{p}}{TP_{p}+FN_{p}} \end{array}$$

## Results

### Patient Basal Information and Characteristics

**Figure 1:**
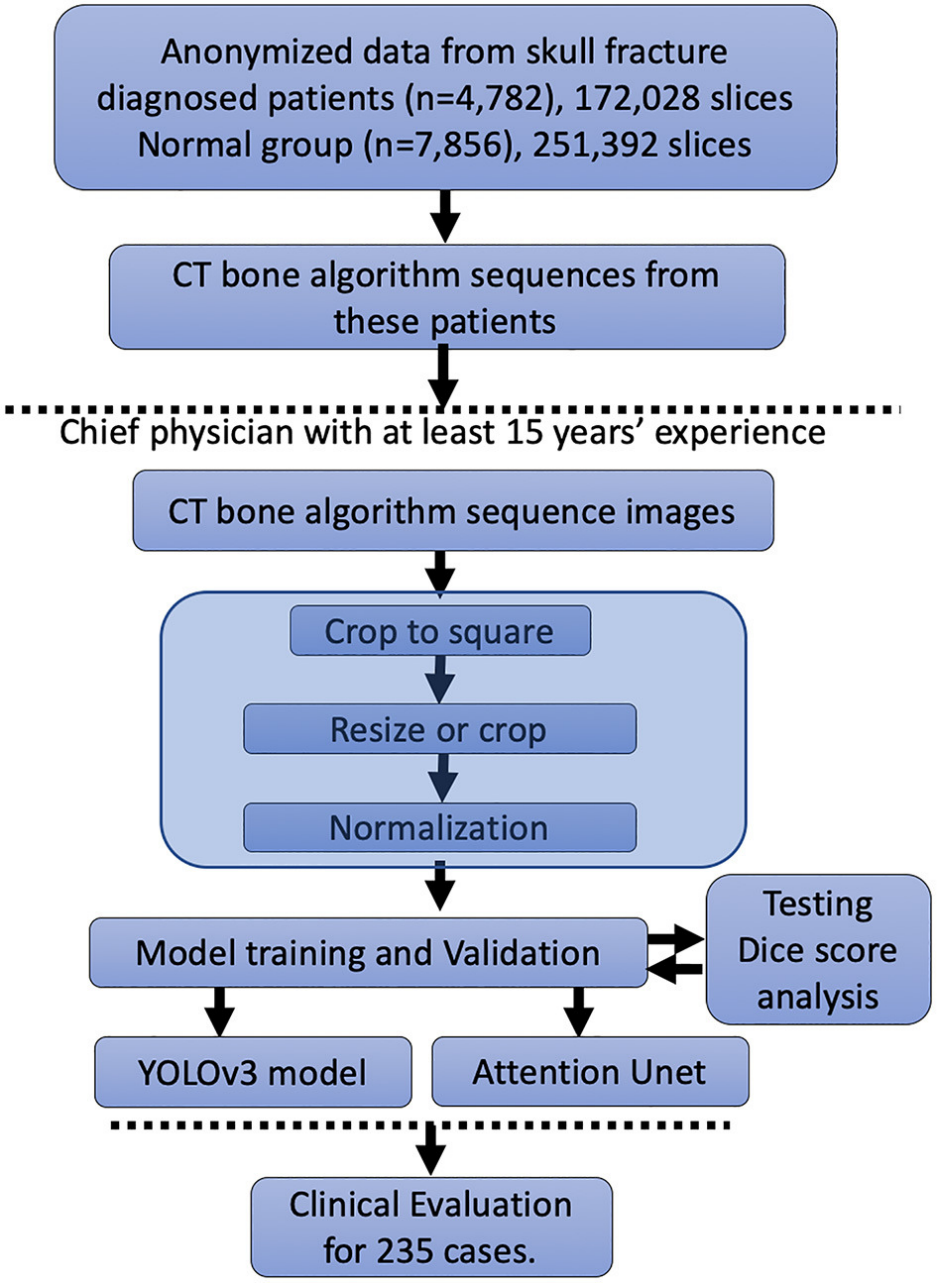
Workflow of the study.

In Figure 1, we represent the entire method for the DLS setup. A total of 172,028 patients' CT bone algorithm sequence images performed in 4,782 skull fracture patients were included. We distributed these data into the training cohort, validation cohort, and testing cohort (data not shown) randomly; thus, no significant differences in sex or age could be observed (data not shown). Additionally, this study included 251,392 slices from 7,856 healthy people.

**Figure 2:**
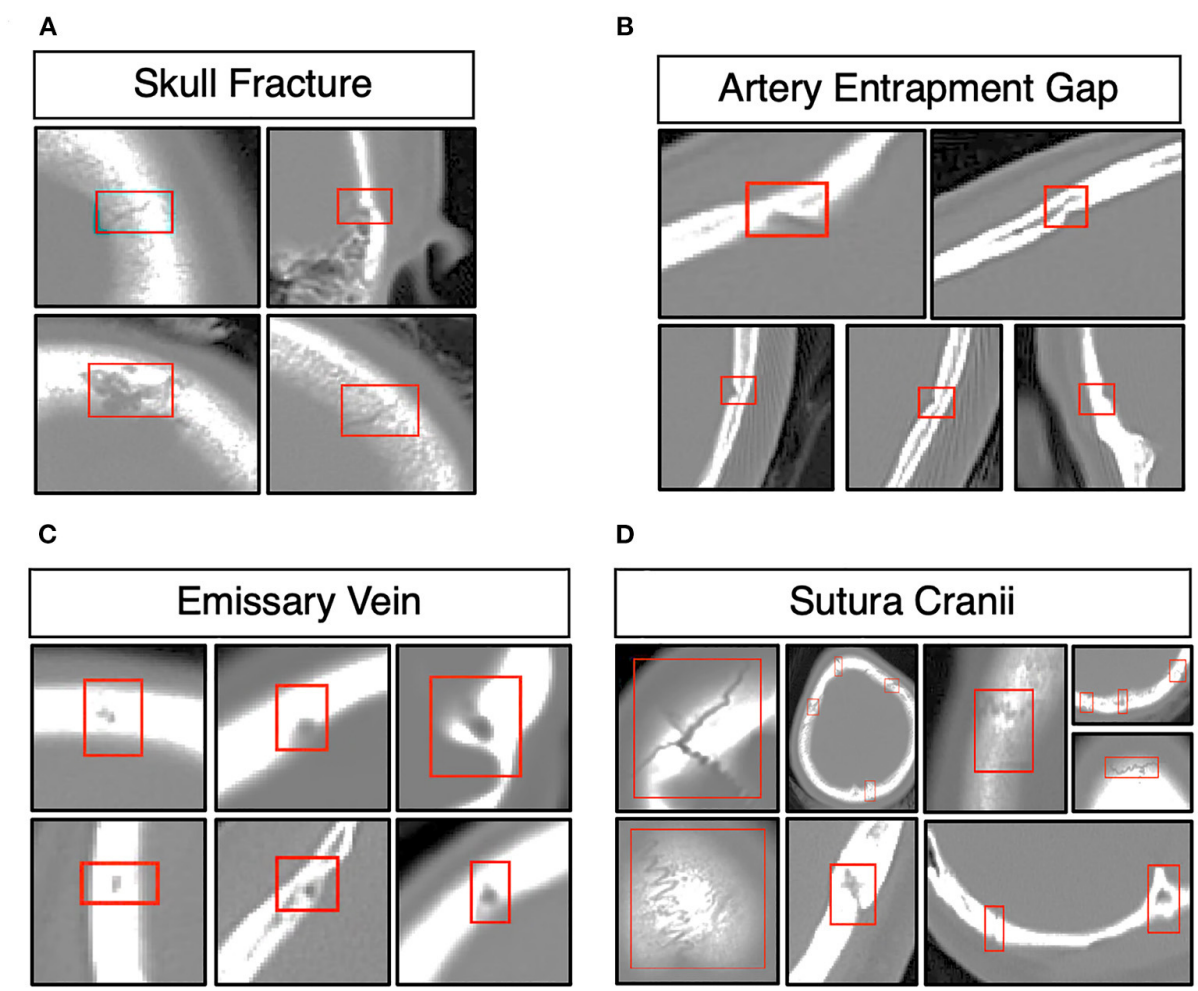
Morphology of skull fractures and structures analogous to skull fractures. **(A)** Morphology of skull fractures in different positions and different fracture morphologies. **(B)** Skull fracture mimics the structure of the artery entrapment gap. **(C)** Skull fracture mimics the structure of the emissary's vein. **(D)** Skull fracture mimics the structure of the sutura crania.

The morphology of the skull fracture and structures analogous to skull fractures, such as the structure of the artery entrapment gap, the structure of the emissary vein, and the structure of the sutura crania, as shown in Figure 2. The distribution of the lesion data has no bias due to the random distribution method. The parameters of these data were obtained from a similar investigator and scanner. We confirmed the scanner parameter by pixel and thinness, the brightness, and contrast data were normalized before being fed into the DLS system.

### Set-Up DLS and Performance of the DLS Contouring

**Figure 3:**
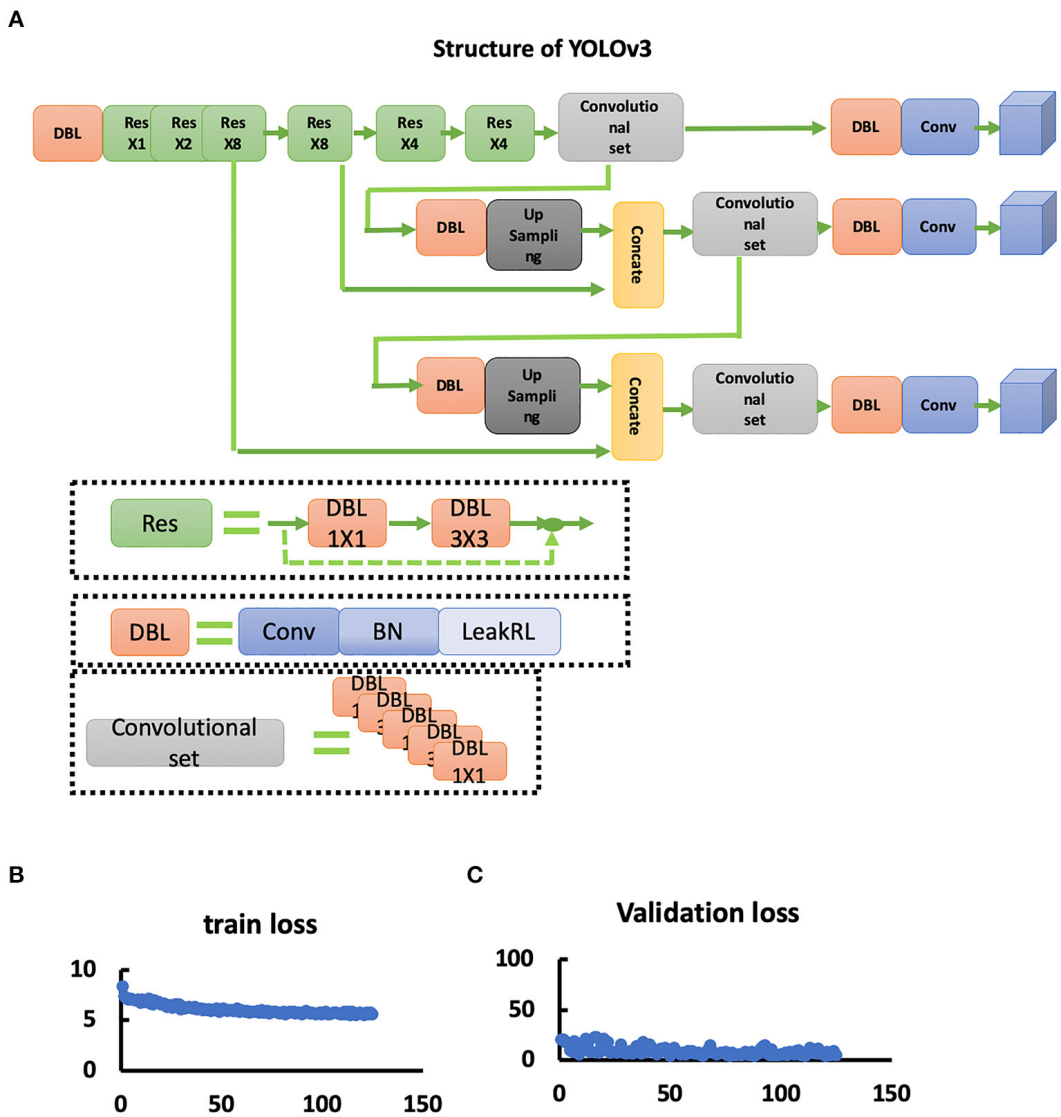
YOLOv3 network. **(A)** Structure of the YOLOv3 network. **(B)** Train loss curve of the YOLOv3 network. **(C)** Validation loss curve of the YOLOv3 network.

**Figure 4:**
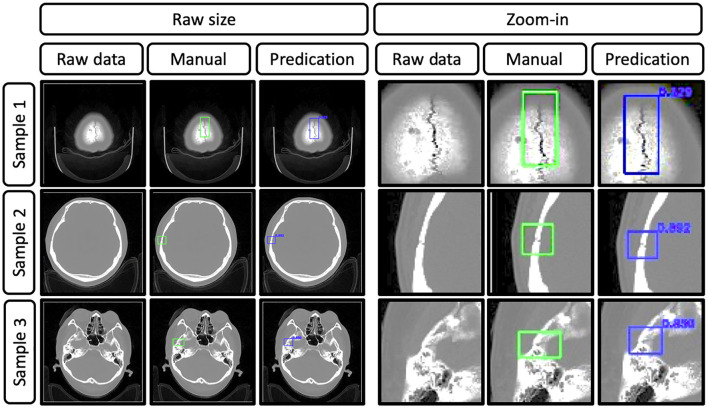
Representative images show the raw data of skull fractures based on CT bone algorithm sequence slices, manual skull fracture labeling, and prediction (by the YOLOv3 network). Samples 1, 2, and 3 were from different patients, and the Dice scores were 0.829, 0.892, and 0.850, respectively.

**Figure 5:**
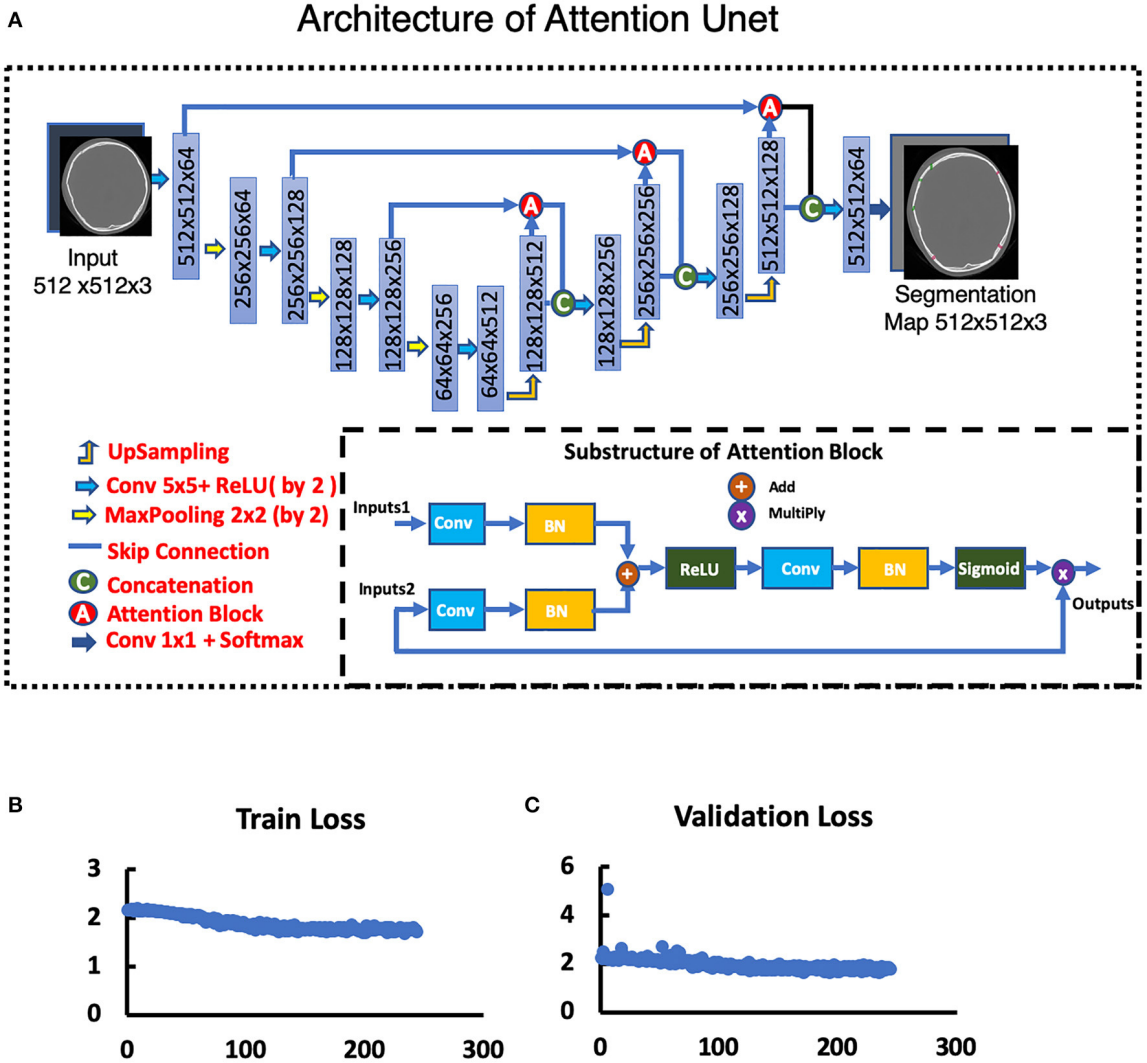
Attention Unet. **(A)** Architecture of Attention Unet. **(B)** Train loss curve of Attention Unet. **(C)** Validation loss curve of Attention Unet.

**Figure 6:**
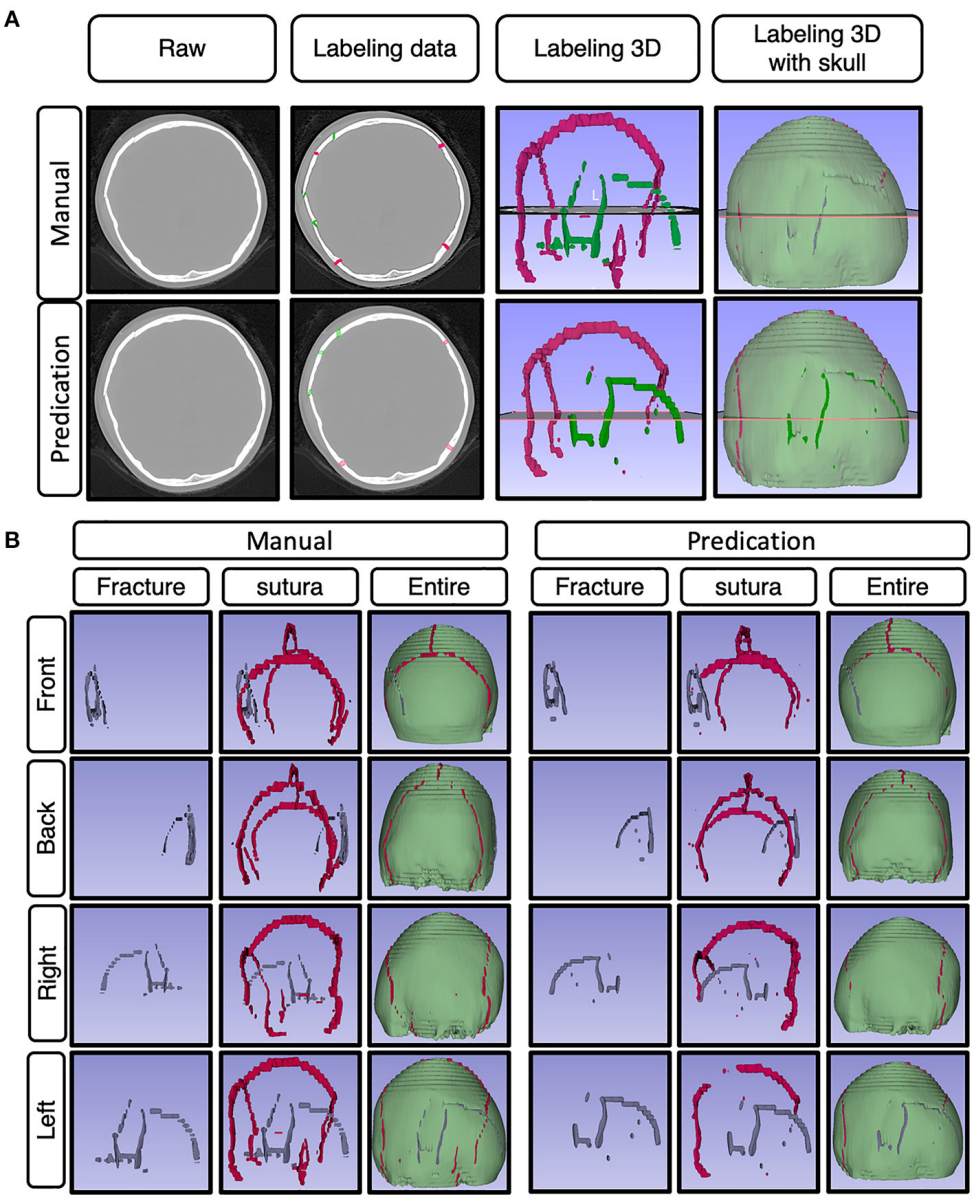
Represented images for the demo case. **(A)** Representative images showing the raw data of skull fractures based on CT bone algorithm sequence slices, manual skull fracture labeling, 3-D reconstruction images of sutura and fracture, and 3-D reconstruction images of sutura, fracture, and whole skull. **(B)** The represented images show the demo case from a 3D perspective (front, back, right side, left side), with manually labeled images and predication-labeled images.

To set up the DLS, the labeling data for the model training and validation were manually performed. In summary, we manually labeled ~172,028 images for training and validation (Figure 1). For the first DLS, named YOLOv3, the proposed 2-dimensional convolutional neural network's network architecture is shown in Figure 3. More detailed information on the network architecture can be found in the Methods of Network Architecture (YOLOv3) section. After training and validation, the DLS was tested using the testing data set. The accuracy of the DLA-generated masking is represented in Figure 4 sample 1 to sample 3 with a Dice score around 0.87. For the second DLS, named Attention Unet, the proposed 2D deep learning segmentation framework's network architecture is shown in Figure 5. More detailed information for the network can be found in the method of network architecture (Attention Unet) section. After training and validation, the DLS was tested using the testing data set. The accuracy of the DLS-generated masking is represented in Figure 6, with a Dice score of 0.72.

### Results on the Test Dataset

Following the training of both deep leering systems for skull fracture identification, their performance was tested on the test dataset containing the CT scans of 235 patients. On this dataset, the object detection approach using YOLOv3 achieved a box-wise precision and recall of 0.894 and 0.587, respectively. The algorithm was able to identify at least one skull fracture layer in the patients, achieving the sensitivity of 80.64% in the identification of patients with skull fractures.

On the same dataset, the segmentation approach of skull fracture identification using the modified attention U-Net architecture achieved a lesion-level precision and recall of 0.71 and 0.567, respectively. The algorithm was able to identify at least one lesion in the patient and achieved a sensitivity of 82.8% in the identification of patients with skull fractures. Despite the relatively low lesion-wise recall, a high degree of visual agreement was observed between the predicted and ground truth skull fracture segmentations. Some examples of fracture segmentation using the modified attention U-net are presented in Figure 6. The complete results on the internal test dataset are presented in Table 1.

**Table 1:** Skull fracture identification on the test dataset.

**Model**		**Precision**	**Recall**
YOLOv3	Box-wise	0.5722	0.7302
Modified attention U-net	Pixel-wise	0.410	0.419
	Lesion-wise	0.710	0.567

### Clinical Evaluation

To understand the generalizability of the model, the performance of the two models was tested on the test dataset of 235 patients. In this dataset, the YOLOv3 architecture was able to identify the presence of skull fracture in 75 of the 93 patients. Also, it correctly predicted 122 of the 142 people to not have any skull fracture. This resulted in patient-level sensitivity, specificity, and accuracy of 80.64, 85.92, and 83.83%, respectively. For the same dataset, the patient level sensitivity, specificity, and accuracy of the modified attention U-net was 82.8, 88.73, and 88.26%, respectively. More detail information of the complete result is presented in Table 2.

**Table 2:** Skull fracture identification on the test dataset.

		**YOLOv3**		**Modified attention U-net**	
		**Predicted label**		**Predicted label**	
		***P***	***N***	***P***	***N***
Actual	Have fracture (*P*)	75	18	77	14
Label	Healthy (*N*)	15	127	16	126
	Sensitivity	83.33%		82.80%	
	Specificity	80.65%		88.73%	
	Accuracy	85.96%		88.26%	

## Discussion

In this study, we found that a deep learning CNN has good performance identifying and detecting skull fractures. The trained CNN model exhibits excellent performance to detect skull fractures. This indicates that the well-trained CNN model makes the automated detection and identification of skull fractures possible. With more data about skull fracture included in the training of the CNN model, we think the deep learning CNN may have similar and even superior diagnostic capability to that of the radiologists.

Previous studies have reported the feasibility of applying CNNs in the analysis of medical images, and promising results were achieved in these studies.

Esteva et al. trained a CNN using a dataset of 129,450 clinical images and found that the CNN demonstrated artificial intelligence capable of classifying skin cancer with a level of competence comparable to dermatologists (21). Kooi T et al. performed a head-to-head comparison between CNN models and radiologists on mammogram reading and found that the CNN network was comparable to certified screening radiologists on a patch level and that there was no significant difference between the network and the readers (13). Hua et al. and Nishio et al. reported that deep learning methods could achieve better discriminative results, were promising in computer-aided diagnosis, and could distinguish lung nodule classifications among benign nodules, primary lung cancer, and metastatic lung cancer at different image sizes using a deep convolutional neural network (14, 15). In addition, CNNs have been used to detect fractures on radiographs and have shown promising results (12–25). Kim and MacKinnon trained the CNN network to recognize wrist fractures on lateral wrist radiographs, and their results showed that the AUC was 0.954, with maximized sensitivity and specificity values of 0.9 and 0.88, respectively (22). Urakawa et al. conducted a study to compare the capacities of the VGG_16 network and orthopedic surgeons in detecting intertrochanteric fractures on radiographs, revealing that the diagnostic performance of the CNN exceeded that of orthopedic surgeons (96 vs. 92%) (26). All previous studies have demonstrated that well-trained CNN models may have comparable capabilities of automated detection and identification of certain features in medical images and may have promising applications in the future.

This study has several limitations. First, the size of the original sample in our dataset was small. More samples will be needed in future studies to reduce overfitting and improve performance because the small sample size might restrict the improvement of the CNN's performance in the training and test procedures. Second, the training and assessment of the diagnostic performance of the CNN models were based on standard plain CT scans of the brain, which may limit the application of this method to a practical scenario. In addition, the algorithms currently in use have technical limitations, which may lead to errors in image analysis.

## Conclusion

The well-trained DLS system exhibited an excellent diagnostic capability in distinguishing skull fractures under limited conditions. It could be a trusted tool for the detection of skull fractures. More image data in different clinical conditions will be needed in future studies to improve CNN performance.

## Data Availability Statement

The original contributions presented in the study are included in the article/supplementary material, further inquiries can be directed to the corresponding author/s.

## Ethics Statement

The studies involving human participants were reviewed and approved by Beijing Tiantan Hospital Ethics Committee. Written informed consent to participate in this study was provided by the participants' legal guardian/next of kin. Written informed consent was obtained from the individual(s) for the publication of any potentially identifiable images or data included in this article.

## Author Contributions

WS, JG, and XMa wrote the initial draft of the manuscript and provided both figures and made preliminary revisions. ZW, ZL, and SW contributed to DLS development and medical test organization. QW, ZW, YW, ZL, XMe, and KH made preliminary revisions to the manuscript. YL, KH, and YW made crucial revisions to the manuscript. All authors planned the manuscript, critically revised the initial draft, and made final improvements prior to submission.

## Funding

This study was funded by the China National Neurological Clinical Research Center, Beijing Postdoctoral Research Foundation (ZZ 2019-09), and China Postdoctoral Science Foundation (No. 2019M660719).

## Conflict of Interest

The authors declare that the research was conducted in the absence of any commercial or financial relationships that could be construed as a potential conflict of interest.

## Publisher's Note

All claims expressed in this article are solely those of the authors and do not necessarily represent those of their affiliated organizations, or those of the publisher, the editors and the reviewers. Any product that may be evaluated in this article, or claim that may be made by its manufacturer, is not guaranteed or endorsed by the publisher.
